# Arthroplasty registries at a glance: an initiative of the International Society of Arthroplasty Registries (ISAR) to facilitate access, understanding, and reporting of registry data from an international perspective

**DOI:** 10.2340/17453674.2024.42706

**Published:** 2025-01-24

**Authors:** Anne LÜBBEKE, Lotje A HOOGERVORST, Perla J MARANG-VAN DE MHEEN, Heather A PRENTICE, Ola ROLFSON, Rob G H H NELISSEN, Arnd STEINBRÜCK, Gearoid MCGAURAN, Christophe BAREA, Kajsa ERIKSON, Alma B PEDERSEN, Martyn PORTER, the ISAR group

**Affiliations:** 1 Division of Orthopaedics and Trauma Surgery, Geneva University Hospitals and University of Geneva, Switzerland; 2 Nuffield Department of Orthopaedics, Rheumatology and Musculoskeletal Sciences, University of Oxford, UK; 3 Department of Orthopaedics, Leiden University Medical Center, The Netherlands; 4 Safety & Security Science, Delft University of Technology, The Netherlands; 5 Medical Device Surveillance & Assessment, Kaiser Permanente, San Diego, CA, USA; 6 Department of Orthopedics, Institute of Clinical Sciences, Sahlgrenska Academy, University of Gothenburg, Gothenburg, Sweden and the Swedish Arthroplasty Register, Gothenburg, Sweden; 7 German Arthroplasty Registry (EPRD), Berlin, Germany; 8 Medical Devices Department, Health Products Regulatory Authority, Dublin, Ireland; 9 Department of Clinical Epidemiology, Aarhus University Hospital, DK and Department of Clinical Medicine, Aarhus University, DK; 10 Emeritus Consultant Orthopaedic Surgeon, Wrightington Hospital, UK; 11 Bristol University, UK

## Abstract

**Background and purpose:**

The amount of information publicly available from arthroplasty registries is large but could be used more effectively. This project aims to improve the knowledge concerning existing registries to facilitate access, transparency, harmonization, and reporting.

**Methods:**

Within the International Society of Arthroplasty Registries (ISAR) we aimed at developing, testing, adopting, and making publicly available a short, standardized registry description with items considered relevant for stakeholders using a cross-sectional study survey. Items were chosen based on a literature review and expert advice, selected by 9 ISAR working group members, tested iteratively in 3 registries, and commented upon by 4 external experts. All 29 ISAR member registries as of July 2023 were invited to participate in the project.

**Results:**

Included items covered general descriptive information regarding registries, information related to governance, outcomes, data quality, data access, and registry production. The template was adopted, completed, and made publicly available by 25 of the 29 registries. Of those, 2/3 were national registries. 23 captured both hip and knee arthroplasties and 10 captured shoulder arthroplasties. Most registries had public reporting of data quality, methods, and results. Data was accessible in all but 2 registries, mainly as aggregated data. Important items relevant to registry quality for researchers to consistently indicate in scientific papers include scope, inclusion criteria, outcomes definitions, coverage/completeness, and validation processes.

**Conclusion:**

This ISAR initiative implemented a short, standardized description to facilitate appropriate use of orthopedic registry data worldwide relevant for a diverse group of stakeholders including researchers, industry, public health and regulatory agencies.

Arthroplasty registries have existed since the 1970s [1]. Their value for stakeholders is well described and includes improving patients’ outcomes and quality of care as well as facilitating research [2-6]. Registries provide information on real-world outcomes on a large scale (e.g., at national level), which is increasingly used by other stakeholders, e.g., for post-market surveillance in regulatory decision-making [7-9] or in scientific publications [10].

The usefulness and usability of registry data depend largely on understanding how the data was obtained, why it was recorded, the quality of data analysis, and its users’ ability to interpret the results [11]. Annual reports and peer-reviewed publications from registries have shown differences regarding incidence and indication for surgery, patient characteristics, implants and fixation methods used, and implant survival. These differences could partly be due to factors such as variations in data collection methods, definitions, and data quality, highlighting the need for harmonization and transparency [12]. In 2005, the International Society of Arthroplasty Registries (ISAR) was established with the mission of “improving outcomes for individuals receiving joint replacement surgery worldwide” [13].

To enhance and facilitate appropriate use of registry data among stakeholders it is important to provide knowledge concerning registries that is publicly available, short, and harmonized across registries internationally. ISAR takes here the initiative to provide “Arthroplasty registries at a glance” and thereby to contribute to better and more effective use including consistent reporting of registry data.

Thus, this project aims to:

1. develop a template for a short, focused, harmonized description of an arthroplasty registry, capturing the key characteristics needed to interpret and use their data;
2. test the template’s content with ISAR participating registries and selected stakeholders;
3. apply the template within the ISAR participating registries; and
4. provide a use case: guidance for researchers regarding scientific reporting from registries.

## Methods

### Study design

This is a cross-sectional survey reporting on the key characteristics of each arthroplasty registry and the STROBE guidelines were followed. We describe the development, testing, and implementation of a short, standardized registry description in English. All ISAR member registries as of July 2023 were invited to participate in the initiative.

### Development and testing of the template

A list of items to be considered for inclusion in the template was identified based on a literature review, previous experience with harmonization in international registry collaboration, and expert advice [11-12,14-17]. The list of items and their definitions was discussed during several ISAR workgroup meetings between March 2021 and March 2023. ISAR workgroup members (n = 9; with combined expertise in orthopedic surgery, arthroplasty registry lead, medical device regulation, and epidemiology), had the opportunity to exclude and include items on the list. The items that were agreed upon by all ISAR workgroup members formed the first draft of the ISAR registries template. After the ISAR registries template draft was made, it was tested by 3 registries (1 national, 1 regional, and 1 hospital-based) for ease of use and understanding and modified accordingly. In the next step the template was reviewed by medical device regulators (n = 2) and regulatory science researchers (n = 2). A revised version, which integrated their feedback, was again discussed during an ISAR workgroup meeting, where the final version of the template was agreed upon (see Supplementary data).

### Data collection using the template and application

Data were collected from the registries using the online application Microsoft Forms (Microsoft Corp, Redmond, WA, USA) and transformed into a CSV file for final descriptive analysis. The completed template was sent back to each registry in a reader-friendly version to be made available by the registry on their website and/or in their annual report.

### Use case: Guidance for scientific reporting of registry data

As an example of how information collected by the template can be used for a specific stakeholder group, in this case researchers, items were selected that should be included in scientific reporting of studies from arthroplasty registries. We showed how these linked to the requirements of the REporting of studies Conducted using Observational Routinely collected health Data (RECORD) [18] by developing an extension to the statement. RECORD itself is an extension of the Strengthening the Reporting of Observational Studies in Epidemiology (STROBE) guidelines [19].

## Results

### Template description

The template included general information on the registry, information on input important to interpret the data, and information on the outputs produced by the registry. More specifically, the following elements (Supplementary data) were considered important and included in the template:

1. general descriptive information (when, where, what, whom to contact);
2. registry input information:
  - information on data ownership, funding, and consent;
  - definition of main outcomes;
  - data quality (coverage, completeness, validation processes, response rates of patient-reported outcome measurements [PROMs]);
  - data access (data linkage and data sharing);
3. registry outputs (outcomes reported, reports and/or publications).

### Data collection using the template

29 registries were identified as members of ISAR in 2023 (Figure). The first established registry was an institution-based registry from the United States of America (USA): the Mayo Clinic registry. The most recently established registry was the South African provider-based registry “JointCare” that started in 20^15^. Of the 29 eligible registries, 25 registries completed the ISAR template and were included in the analysis. Most registries were national (18 out of 25), and the remainder were regional (4), hospital-based (2), or provider-based (1) (Figure and Table 1). Data was owned either by a public authority (10 out of 25), a healthcare provider/institution (9 out of 25), or by the relevant national orthopedic society (6 out of 25). Most registries reported that they captured data on both hip and knee arthroplasties (23 out of 25), followed by shoulder arthroplasties (10), and a few registries captured data on ankle, elbow, spine, and hand implants (Table 1). 3 registries reported capturing all arthroplasty implants: the Australian, New Zealand, and Norwegian registries.

**Table 1:**
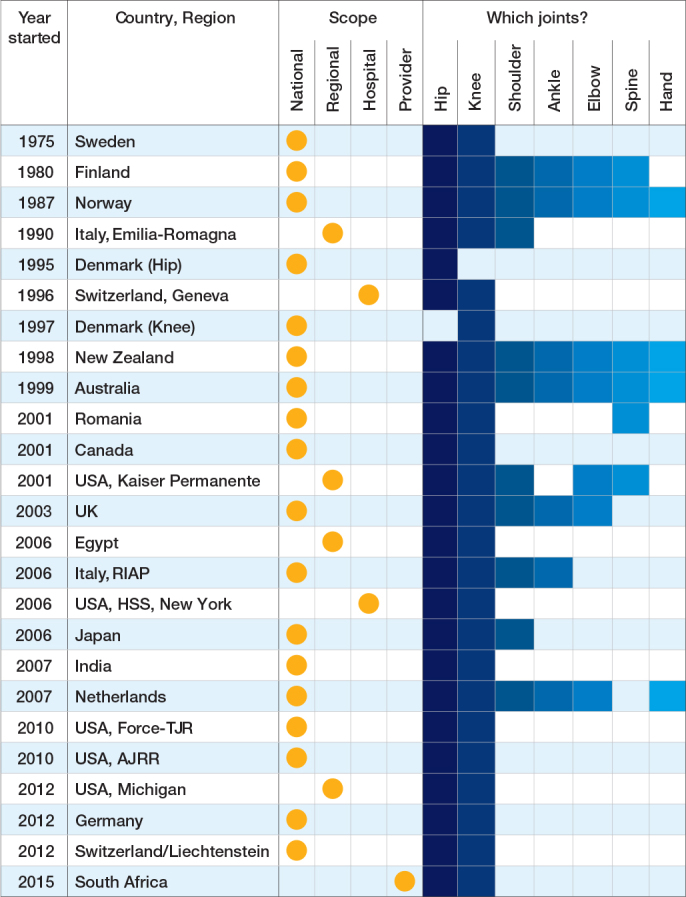
Scope of registries and types of joint arthroplasties covered

**Figure:**
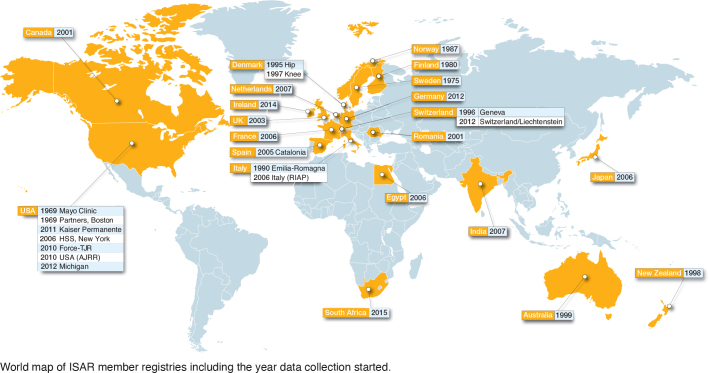


Hospital coverage (the number of participating hospitals relative to the total number of eligible hospitals) ranged from 12% in 1 registry to 100% in 14 registries and was unknown for 1 registry (Table 2). In general, hospital coverage increased with the number of years the registry had existed. Completeness of primary hip/knee procedures captured by the registry was ≥ 95% for 17 and ≥ 80% for 19 registries. Validation of completeness against an external data source was performed by 23 registries. Regarding outcomes reported by registries, revision for any cause was the most frequently reported outcome (n = 23) followed by PROs at n = 15. Specific reasons for revision were captured by 22 registries, and Unique Device Identifiers (UDIs) by 10 registries. Implant outlier identification procedures (i.e., procedures to identify implants with significantly higher risks than other comparable implants) were implemented by approximately half of the registries (n = 13). Of those 13, 8 reported the outlier implants publicly. Sharing registry data for research purposes with external parties was mostly possible, for anonymized patient-level data under specific conditions as well as for aggregated data (19 and 23, respectively) (Table 2). Publicly available annual reports were produced by 21 registries (Table 3). The completed individual registries’ templates are available as Supplementary data.

**Table 2:**
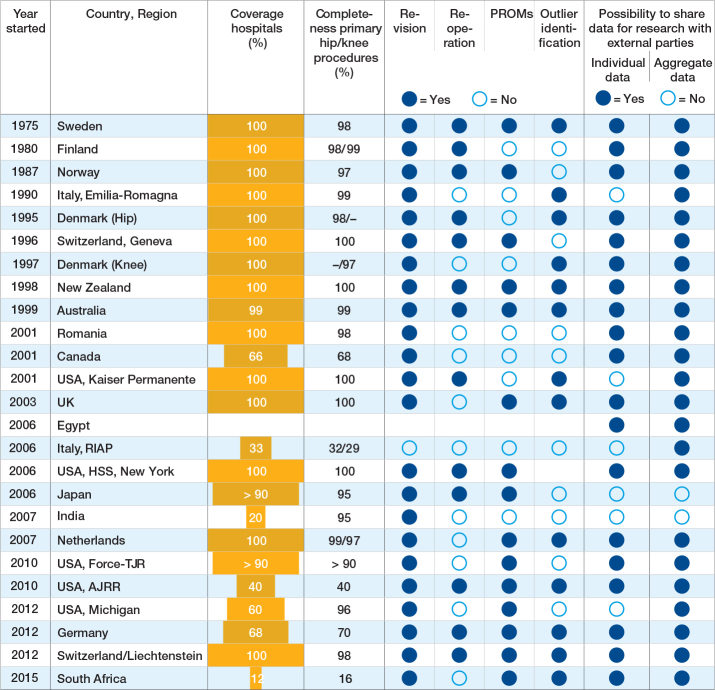
Overview of coverage, procedure completeness, outcomes captured, and data sharing

**Table 3:** Link to registries’ online reports

Country/Region	Registry name	Annual report website
Sweden	Swedish Arthroplasty Register	sar.registercentrum.se/about-the-register/annual-reports/p/SJW4-ZGyo
Finland	Finnish Arthroplasty Register	thl.fi/far
Norway	Norwegian Arthroplasty Register (NAR)	helse-bergen.no/nrl
Italy, Emilia-Romagna	Register of Orthopaedic Prosthetic Implants (RIPO)	ripo.cineca.it/authzssl/Reports.html
Denmark	Danish Hip Arthroplasty Register	dhr.dk
Switzerland, Geneva	Geneva Arthroplasty Registry (GAR)	Available upon request
Denmark	Danish Knee Arthroplasty Registry	www.sundhed.dk/sundhedsfaglig/kvalitet/kliniske-kvalitetsdatabaser/planlagt-kirugi/knaealloplastikregister/
New Zealand	New Zealand Joint Registry	www.nzoa.org.nz/nzoa-joint-registry
Australia	Australian Orthopaedic Association National Joint Replacement Registry	aoanjrr.sahmri.com/annual-reports-2022
Romania	Romanian Arthroplasty Register	www.rne.ro
Canada	Canadian Joint Replacement Registry	www.cihi.ca/en/cjrr-annual-report-hip-and-knee-replacements-in-canada
USA, Kaiser Permanente	Kaiser Permanente Medical Device Surveillance and Assessment/National Implant Registries	annualreport.kpimplantregistries.org/annual-report/
UK	National Joint Registry (NJR)	reports.njrcentre.org.uk/
Egypt	Egyptian Community Arthroplasty Registry	None currently available
Italy	Italian Arthroplasty Registry (RIAP)	riap.iss.it/riap/en/activities/reports/
USA, HHS, New York	Hospital for Special Surgery	None currently available
Japan	Japanese Orthopaedic Association National Registry/Japan Arthroplasty Register	https://www.joanr.org/ and https://jsra.info/
India	Indian Society of Hip & Knee Surgeons	www.ishks.com
Netherlands	Dutch Arthroplasty Register (LROI)	www.lroi-report.nl
USA, Force-TJR	Function and Outcomes Research for Comparative Effectiveness in Total Joint Replacement (FORCE-TJR)	Available upon request
USA, AJRR	American Joint Replacement Registry	aaos.org/registries
USA, Michigan	Michigan Arthroplasty Registry Collaborative Quality Initiative (MARCQI)	marcqi.org/marcqi-registry-reports-marcqi-annual-reports/
Germany	German Arthroplasty Registry (EPRD)	www.eprd.de/en/downloads/reports
Switzerland/Liechtenstein	SIRIS—Swiss National Joint Registry Hip & Knee	www.siris-implant.ch/
South Africa	JointCare Registry	www.joint-care.co.za

### Use of the template by researchers

As data from arthroplasty registries are used extensively by researchers, they are important for this group of stakeholders. Table 4 (see Appendix) shows the items that should consistently be reported—as an extension of the STROBE and RECORD checklists—in publications of arthroplasty-based research studies to better understand the data quality and interpret strengths and limitations of study findings.

**Table 4:** Template items to be reported in observational studies using arthroplasty registry data

	Item no.	STROBE checklist	RECORD items	Template items
**Title and abstract**				
	1	(a) Indicate the study’s design with a commonly used term in the title or the abstract. (b) Provide in the abstract an informative and balanced summary of what was done and what was found	RECORD 1.1: The type of data used should be specified in the title or abstract. When possible, the name of the databases used should be included. RECORD 1.2: If applicable, the geographic region and time frame within which the study took place should be reported in the title or abstract. RECORD 1.3: If linkage between databases was conducted for the study, this should be clearly stated in the title or abstract	1.1: The name of the registry and the joint(s) replaced should be included in the title and abstract when possible
**Introduction**				
**Background rationale**	2	Explain the scientific background and rationale for the investigation being reported		
**Objectives**	3	State specific objectives, including any prespecified hypotheses		
**Methods**				
**Study Design**	4	Present key elements of study design early in the paper		
**Setting**	5	Describe the setting, locations, and relevant dates, including periods of recruitment, exposure, follow-up, and data collection		5.1: Describe the scope of the registry (e.g., national, regional, institutional, multicentric, provider-based), the inclusion criteria, and the year data collection started Provide a reference to the registry’s website when possible
**Participants**	6	(a) Cohort study: Give the eligibility criteria and the sources and methods of selection of participants Describe methods of follow-up. Case-control study: Give the eligibility criteria and the sources and methods of case ascertainment and control selection. Give the rationale for the choice of cases and controls. Cross-sectional study: Give the eligibility criteria and the sources and methods of selection of participants (b) Cohort study: For matched studies, give matching criteria and number of exposed and unexposed. Case-control study: For matched studies, give matching criteria and the number of controls per case	RECORD 6.1: The methods of study population selection (such as codes or algorithms used to identify subjects) should be listed in detail. If this is not possible, an explanation should be provided RECORD 6.2: Any validation studies of the codes or algorithms used to select the population should be referenced. If validation was conducted for this study and not published elsewhere, detailed methods and results should be provided RECORD 6.3: If the study involved linkage of databases, consider use of a flow diagram or other graphical display to demonstrate the data linkage process, including the number of individuals with linked data at each stage	
**Variables**	7	Clearly define all outcomes, exposures, predictors, potential confounders, and effect modifiers. Give diagnostic criteria, if applicable	RECORD 7.1: A complete list of codes and algorithms used to classify exposures, outcomes, confounders, and effect modifiers should be provided. If these cannot be reported, an explanation should be provided	7.1: If the study involves specific implants the source(s) of implant details should be provided (e.g., catalogue number, unique device identifier) 7.2: If the study assesses revision or reoperation a definition should be provided including for which causes they were performed (e.g., all-cause revision) and at which date the study follow-up ended
**Data sources/measurement**	8	For each variable of interest, give sources of data and details of methods of assessment (measurement). Describe comparability of assessment methods if there is more than 1 group		8.1: Coverage of hospital registration and completeness of procedure registration should be indicated for the period relevant to the study 8.2: Data source(s) for validation of coverage and completeness should be indicated 8.3: If patient-reported outcomes (PROs) are reported indicate the response rate (or completeness) for each instrument at each time point of interest for the study. Discuss implications of low response rate
**Bias**	9	Describe any efforts to address potential sources of bias		
**Study size**	10	Explain how the study size was arrived at		
**Quantitative variables**	11	Explain how quantitative variables were handled in the analyses. If applicable, describe which groupings were chosen and why		
**Statistical methods**	12	(a) Describe all statistical methods, including those used to control for confounding. (b) Describe any methods used to examine subgroups and interactions. (c) Explain how missing data were addressed. (d) Cohort study: If applicable, explain how loss to follow-up was addressed. Case-control study: If applicable, explain how matching of cases and controls was addressed. Cross-sectional study: If applicable, describe analytical methods taking account of sampling strategy. (e) Describe any sensitivity analyses		
**Data access and cleaning methods**		N/A	RECORD 12.1: Authors should describe the extent to which the investigators had access to the database population used to create the study population RECORD 12.2: Authors should provide information on the data-cleaning methods used in the study	
**Linkage**		N/A	RECORD 12.3: State whether the study included person-level, institutional-level, or other data linkage across 2 or more databases The methods of linkage and methods of linkage quality evaluation should be provided	12.1: State if the study includes data obtained through linkage with (an)other database(s) The name of the database(s), the additional variables obtained, and the method of linkage should be indicated
**Results**				
**Participants**	13	(a) Report the numbers of individuals at each stage of the study (e.g., numbers potentially eligible, examined for eligibility, confirmed eligible, included in the study, completing follow-up, and analyzed). (b) Give reasons for nonparticipation at each stage. (c) Consider use of a flow diagram	RECORD 13.1: Describe in detail the selection of the persons included in the study (i.e., study population selection), including filtering based on data quality, data availability, and linkage. The selection of included persons can be described in the text and/or by means of the study flow diagram	
**Descriptive data**	14	(a) Give characteristics of study participants (e.g., demographic, clinical, and social) and information on exposures and potential confounders. (b) Indicate the number of participants with missing data for each variable of interest. (c) Cohort study: summarize follow-up time (e.g., average and total amount)		
**Outcome data**	15	Cohort study: Report numbers of outcome events or summary measures over time. Case-control study: Report numbers in each exposure category or summary measures of exposure. Cross-sectional study: Report numbers of outcome events or summary measures		
**Main results**	16	(a) Give unadjusted estimates and, if applicable, confounder-adjusted estimates and their precision (e.g., 95% confidence interval) Make clear which confounders were adjusted for and why they were included. (b) Report category boundaries when continuous variables were categorized. (c) If relevant, consider translating estimates of relative risk into absolute risk for a meaningful time period		
**Other analyses**	17	Report other analyses done—e.g., analyses of subgroups and interactions and sensitivity analyses		
**Discussion**				
**Key results**	18	Summarize key results with reference to study objectives		
**Limitations**	19	Discuss limitations of the study, taking into account sources of potential bias or imprecision. Discuss both direction and magnitude of any potential bias	RECORD 19.1: Discuss the implications of using data that were not created or collected to answer the specific research question(s) Include discussion of misclassification bias, unmeasured confounding, missing data, and changing eligibility over time, as they pertain to the study being reported	
**Interpretation**	20	Give a cautious overall interpretation of results considering objectives, limitations, multiplicity of analyses, results from similar studies, and other relevant evidence		
**Generalizability**	21	Discuss the generalizability (external validity) of the study results		
**Other information**				
**Funding**	22	Give the source of funding and the role of the funders for the present study and, if applicable, for the original study on which the present article is based		
**Accessibility of protocol, raw data, and programming code**		N/A	RECORD 22.1: Authors should provide information on how to access any supplemental information such as the study protocol, raw data, or programming code	

N/A: not applicable.

## Discussion

We developed a template for a short, focused, harmonized description of an arthroplasty registry, capturing the key characteristics needed to interpret and use their data. After testing its content, which was based on input from diverse registry stakeholder groups, in 3 different types of registries, the template was filled in and adopted by the vast majority of the registries.

We also provided a use case in the form of a list of items that should be reported in scientific publications based on arthroplasty registries. The items include the registry’s scope, inclusion criteria, outcomes definitions, coverage/completeness, and validation processes.

Taken together, the cross-sectionally obtained information from the templates shows that ISAR member registries monitor the outcomes of arthroplasty procedures in all continents, but with a larger concentration in Europe and North America, and that the monitoring focuses mainly on risks but in some cases also on benefits as measured with use of patient-reported outcomes. 2/3 of the registries operate on a national level, and half of them have been active for more than 2 decades. In the majority, data quality with respect to coverage and completeness is high, methods and results are reported transparently, and aggregated or individual data is accessible. Overall, the amount of publicly available information produced for stakeholders is extensive, but this information is underutilized by interested parties, especially in the regulatory field [20], and reporting of registry information in scientific publications is inconsistent.

This initiative to standardize information across ISAR member registries intends to increase the discoverability, accessibility, interpretability, and usability of registry data, by creating and implementing a short, standardized template to describe registries, thereby increasing their more effective use. For stakeholders who are interested in a specific arthroplasty implant—such as regulators, clinicians, notified bodies, and industry personnel—this template can help them to more easily identify all registries that collect data on that implant as well as outcomes. Moreover, this initiative has the objective to strengthen individual registries’ visibility and aims, and to further harmonize data input and output and increase quality and usability of the registries’ work. For researchers using registry data, the benefit is to facilitate consistent reporting within the RECORD checklist. The completed templates will be made available by each registry on their websites and/or at the beginning of their annual reports, and on the ISAR website (https://www.isarhome.org/). The templates will be updated annually.

Prior publications have either described general requirements with respect to the structure, quality, analysis, and use of medical device registries [11,15,16] or they have focused on specific medical specialties and areas, such as the scope, content, and quality of orthopedic and cardiovascular registries [12,21], the use of PROs in arthroplasty [22], or benchmarking and outlier identification in arthroplasty registries [23].

### Strengths and limitations

In our work, for the first time, we developed, tested, and implemented a short-form template (“Arthroplasty registries at a glance”) in a specific society (ISAR) bringing together a particular group of arthroplasty registries. The current initiative covers the registries that were members of ISAR and agreed to participate in 2023. Participation rate of the members was high (25 out of 29). Nevertheless, in addition to any changes that may be needed in the existing template, it is essential that continued efforts are made, coordinated by ISAR, to ensure that new registries will be included and encouraged to adopt the template. Moreover, registries covering the spine tend to be more diverse and are often run by more than 1 medical society, thus these results may give only a partial view of existing spine registries worldwide. For the template to cover a more diverse range of medical device registries, some modifications will be necessary such as adding items regarding patient inclusion and exclusion criteria and adapting the main outcomes. Finally, while we included all ISAR members at a certain point in time there are a few other registries (n = 6) we are aware of that are not included in this work for reasons related to early phase of registry creation or pending ISAR membership.

### Conclusion

We developed, tested, and implemented a template for a short, standardized description of ISAR member registries. The included items cover descriptive information and main registry input and output including outcome measures, information related to governance, data quality, data access, and registry production, which were identified as relevant elements for a diverse group of stakeholders. We also showed, as an extension to the RECORD checklist, how these items can be used in scientific reporting of studies using registry data. The template could be the step forward to improve harmonization, quality, interpretability, and usability of registry data, thereby allowing for a more effective and appropriate use by interested parties.

## Supplementary data

Conflict of interest statements, STROBE checklist, ISAR template, list of ISAR members, and complete short descriptions of the 25 registries that participated are available as supplementary data on the article page, doi: 10.2340/17453674.2024.42706

## Notes

The authors would like to thank Tom Melvin, Petra Schnell-Inderst, and Robert Geertsma for their comments on earlier versions of the template and Uwe Otte for the realization of the figures and tables.

Handling co-editor: Bart A Swierstra

*Acta* thanks Ian Harris, Wierd P Zijlstra, and an anonymous reviewer for help with peer review of this manuscript.
